# Efeito Cardioprotetor do Exercício Resistido no Remodelamento Ventricular Esquerdo Associado à Hipertensão Arterial Pulmonar Induzida por Monocrotalina

**DOI:** 10.36660/abc.20220638

**Published:** 2022-10-05

**Authors:** Silvio A. Oliveira-Junior, Alex Y. Ogura, Marianna R. Carvalho, Paula F. Martinez

**Affiliations:** 1 Universidade Federal de Mato Grosso do Sul Campo Grande MS Brasil Universidade Federal de Mato Grosso do Sul (UFMS), Campo Grande , MS – Brasil

**Keywords:** Coração, Hipertensão Arterial Pulmonar, Exercício Físico, Contração Miocárdica

A hipertensão arterial pulmonar (HAP) é caracterizada por resistência vascular pulmonar progressiva, acometendo várias artérias e arteríolas. As alterações estão associadas ao aumento da pós-carga e remodelação do ventrículo direito, caracterizada por hipertrofia grave, inicialmente adaptativa e derivada do gradiente de pressão vascular. ^1 , 2^ Posteriormente, essas alterações são acompanhadas de dilatação do ventrículo direito e função contrátil prejudicada, resultando em fração de ejeção reduzida e insuficiência ventricular. ^3 , 4^ Clinicamente, as alterações do tamanho e dos parâmetros de função do ventrículo direito têm reconhecida associação com mau prognóstico na hipertensão arterial pulmonar. ^4^

Durante o desenvolvimento da HAP, quando a pressão de enchimento do ventrículo direito está aumentada e o débito cardíaco se deteriora, há concomitante sobrecarga volumétrica e pressórica no ventrículo direito. ^4^ Esses efeitos fazem com que o septo interventricular se desloque para o lado esquerdo, sustentando um movimento paradoxal. Em seguida, a fração de ejeção do ventrículo esquerdo (VE) e o desempenho diastólico são afetados, repercutindo em comprometimento do enchimento diastólico precoce, redução do volume diastólico final e remodelamento adverso. ^2 , 4^ Portanto, a disfunção do VE configura um efeito secundário e importante do desenvolvimento de HAP. ^1 , 2 , 5^

Em termos de tratamento, várias intervenções farmacológicas têm sido adotadas como opções terapêuticas para HAP. Apesar disso, a HAP tem sido associada a uma alta prevalência de mortalidade e morbidade por complicações cardíacas. Geralmente, os pacientes com HAP apresentam astenia, fadiga, dispneia e escores ruins de tolerância ao esforço e qualidade de vida. ^6 , 7^

O treinamento físico é uma ferramenta não farmacológica em potencial para ser utilizada como opção terapêutica contra doenças e complicações cardiovasculares. ^1 , 7^ Vários protocolos de treinamento físico têm sido utilizados como intervenções promissoras em experimentos com HAP. Protocolos de exercícios aeróbicos contínuos promoveram efeitos benéficos no remodelamento do ventrículo direito e da artéria pulmonar. ^8 - 10^ Da mesma forma, o treinamento intervalado de alta intensidade (HIIT) atenuou a pressão sistólica e o remodelamento do ventrículo direito e reduziu a resistência pulmonar total em um modelo experimental de HAP ^11^ induzida por monocrotalina (MCT). Por outro lado, os potenciais impactos das intervenções de treinamento físico sobre os aspectos do VE são pouco esclarecidos em condições experimentais de hipertensão arterial pulmonar.

Na atual edição dos Arquivos Brasileiros de Cardiologia, Soares et al. ^12^ analisaram a influência do treinamento físico resistido na remodelação do VE e no desempenho dos cardiomiócitos em ratos durante o desenvolvimento de HAP induzida por monocrotalina (MCT). Neste elegante estudo, os autores constataram que o exercício resistido aumentou progressivamente a tolerância ao esforço físico durante o desenvolvimento da HAP em ratos submetidos a duas injeções de MCT (20 mg/kg) intervaladas por sete dias. Em comparação aos controles, os animais HAP treinados exibiram sinais de insuficiência cardíaca mais tardiamente. Da mesma forma, o treinamento resistido melhorou a fração de ejeção do VE, as velocidades de contração e de relaxamento de cardiomiócitos. Essas melhorias foram acompanhadas por redução do colágeno tipo I e aumento da quantidade de colágeno tipo III em amostras de VE de animais HAP treinados. As fibras de colágeno miocárdico apresentam diferenças biomecânicas distintas; fibras de colágeno I conferem maior rigidez, enquanto o colágeno tipo III está associado a maior suscetibilidade à deformação mecânica, ^13 , 14^ o que pode estar relacionado a melhor desempenho contrátil do VE.

Portanto, o treinamento físico resistido de baixa a moderada intensidade tem efeitos adjuvantes e cardioprotetores no controle do remodelamento do VE secundário à HAP induzida por MCT. Com base nisso, intervenções semelhantes podem efetivamente minimizar as complicações cardíacas associadas à HAP. Por outro lado, como os parâmetros do treinamento físico variam e podem sustentar múltiplos protocolos, é necessário caracterizar melhor as demandas relativas à velocidade e intensidade, como também é discutido pelos autores, ^12^ além de frequência e duração. Novos estudos contribuirão para elucidar os efeitos de diversos protocolos de treinamento resistido sobre distúrbios cardiopulmonares derivados da HAP induzida por MCT.
