# Supplementary material for: Efeito Cardioprotetor do Exercício Resistido no Remodelamento Ventricular Esquerdo Associado à Hipertensão Arterial Pulmonar Induzida por Monocrotalina
Source: Arq Bras Cardiol. 2022 Oct 5;119(4):585–6. [Article in Portuguese] doi: 10.36660/abc.20220638 (PMC9563880; doi:10.36660/abc.20220638)
Supplement: Translation

# Translation: Cardioprotective Effect of Resistance Exercise on Left Ventricular Remodeling Associated with Monocrotaline-Induced Pulmonary Arterial Hypertension

**Authors:** Silvio A. Oliveira-Junior, Alex Y. Ogura, Marianna R. Carvalho, Paula F. Martinez

**Affiliations:** 1 Brazil Universidade Federal de Mato Grosso do Sul (UFMS), Campo Grande, MS – Brazil

**Keywords:** Heart, Pulmonary Arterial Hypertension, Exercise, Myocardial Contraction

Pulmonary arterial hypertension (PAH) is characterized by progressive pulmonary vascular resistance, affecting several arteries and arterioles. These changes are associated with increased right ventricular afterload and remodeling, characterized by severe hypertrophy, initially adaptive and derived from vascular pressure gradient.^1 , 2^ Afterward, these alterations are accompanied by right ventricular dilation and impaired contractile performance, resulting in reduced ejection fraction and ventricular failure.^3 , 4^ Clinically, right ventricular size and function parameters alterations have a recognized association with bad prognosis in pulmonary arterial hypertension.^4^

During the PAH development, when right ventricular filling pressure has been increased, and cardiac output is deteriorating, there are concomitant volumetric and pressoric overload into the right ventricle.^4^ These effects make the interventricular septum move to the left side, sustaining a paradoxical movement. Next, left ventricle (LV) ejection fraction and diastolic performance are affected, facing impaired early diastolic filling, reduced end-diastolic volume, and adverse remodeling.^2 , 4^ Therefore, LV dysfunction configures a secondary and important effect from PAH onset.^1 , 2 , 5^

In terms of treatment, several pharmacological interventions have been adopted as therapeutic options for PAH. Despite this, PAH condition has been associated with a high prevalence of mortality and morbidity due to cardiac complications. Generally, PAH patients exhibit asthenia, fatigue, dyspnea, and poor scores of effort tolerance and quality of life.^6 , 7^

Physical exercise training is a potential non-pharmacological tool to be used as a therapeutic option for cardiovascular diseases and complications.^1 , 7^ Several physical training protocols have been used as promissory interventions in PAH experiments. Continuous aerobic exercise protocols promoted beneficial effects in the right ventricle and pulmonary artery remodeling.^8 - 10^ Likewise, high-intensity interval training (HIIT) attenuated right ventricle systolic pressure and remodeling and lowered total pulmonary resistance in a rat model of monocrotaline (MCT)-induced PAH.^11^ On the other hand, the potential impacts of exercise training interventions on LV aspects are few clarified in experimental conditions of pulmonary arterial hypertension.

In the current edition of the *Arquivos Brasileiros de Cardiologia* , Soares et al.^12^ analyzed the influence of resistance exercise training on LV remodeling and cardiomyocyte performance in rats during the development of monocrotaline (MCT)-induced PAH. In this elegant study, the Authors found that resistance exercise progressively increased tolerance to physical effort during the development of PAH in rats submitted to two injections of MCT (20 mg/kg) interspaced over seven days. Compared to control counterparts, trained PAH-animals exhibited later-onset heart failure signals. Likewise, resistance exercise training improved LV ejection fraction, cardiomyocyte contraction, and relaxation velocities. These improvements were accompanied decreased amount of type I collagen and increased type III collagen in LV samples from trained PAH-animals. Myocardial collagen fibers have distinct biomechanical differences; collagen I fibers confer higher stiffness, while type III collagen is associated with increased susceptibility to mechanical deformation,^13 , 14^ which could be related to better LV contractile performance.

Therefore, low- to moderate-intensity resistance exercise training has adjuvant and cardioprotective effects in controlling LV remodeling secondary to MCT-induced PAH. Based on this, similar interventions may effectively minimize cardiac complications associated with PAH. On the other hand, as exercise training parameters vary and may sustain multiple protocols, it is necessary to better characterize demands relative to speed and intensity, as also discussed by the authors,^12^ besides frequency and duration. Further studies will contribute to elucidating the effects of diverse resistance exercise training protocols on cardiopulmonary disorders derived from MCT-induced PAH.
